# High prevalence of circulating DS-1-like human rotavirus A and genotype diversity in children with acute gastroenteritis in Thailand from 2016 to 2019

**DOI:** 10.7717/peerj.10954

**Published:** 2021-02-26

**Authors:** Siripat Pasittungkul, Fajar Budi Lestari, Jiratchaya Puenpa, Watchaporn Chuchaona, Nawarat Posuwan, Jira Chansaenroj, John Mauleekoonphairoj, Natthinee Sudhinaraset, Nasamon Wanlapakorn, Yong Poovorawan

**Affiliations:** 1 Faculty of Medicine, Chulalongkorn University, Center of Excellence in Clinical Virology, Bangkok, Bangkok, Thailand; 2 Department of Bioresources Technology and Veterinary, Vocational College, Universitas Gadjah Mada, Yogyakarta, Indonesia; 3 Faculty of Graduate School, Chulalongkorn University, Inter-Department of Biomedical Sciences, Bangkok, Bangkok, Thailand; 4 Faculty of Medicine, Chulalongkorn University, Division of Academic Affairs, Bangkok, Bangkok, Thailand

**Keywords:** Rotavirus A, DS-1-like, Acute gastroenteritis

## Abstract

**Background:**

Human rotavirus A (RVA) infection is the primary cause of acute gastroenteritis (AGE) in infants and young children worldwide, especially in children under 5 years of age and is a major public health problem causing severe diarrhea in children in Thailand. This study aimed to investigate the prevalence, genotype diversity, and molecular characterization of rotavirus infection circulating in children under 15 years of age diagnosed with AGE in Thailand from January 2016 to December 2019.

**Methods:**

A total of 2,001 stool samples were collected from children with gastroenteritis (neonates to children <15 years of age) and tested for RVA by real-time polymerase chain reaction (RT-PCR). Amplified products were sequenced and submitted to an online genotyping tool for analysis.

**Results:**

Overall, 301 (15.0%) stool samples were positive for RVA. RVA occurred most frequently among children aged 0-24 months. The seasonal incidence of rotavirus infection occurred typically in Thailand during the winter months (December-March). The G3P[8] genotype was identified as the most prevalent genotype (33.2%, 100/301), followed by G8P[8] (10.6%, 32/301), G9P[8] (6.3%, 19/301), G2P[4] (6.0%, 18/301), and G1P[6] (5.3%, 16/301). Uncommon G and P combinations such as G9P[4], G2P[8], G3P[4] and G3P[9] were also detected at low frequencies. In terms of genetic backbone, the unusual DS-1-like G3P[8] was the most frequently detected (28.2%, 85/301), and the phylogenetic analysis demonstrated high nucleotide identity with unusual DS-1-like G3P[8] detected in Thailand and several countries.

**Conclusions:**

A genetic association between RVA isolates from Thailand and other countries ought to be investigated given the local and global dissemination of rotavirus as it is crucial for controlling viral gastroenteritis, and implications for the national vaccination programs.

## Introduction

Rotavirus (RV) is the most common etiological agent associated with severe gastroenteritis in children worldwide. Although vaccinations for RV had been licensed for over a decade, RV infection was still responsible for an estimated 128,500 deaths worldwide among children younger than age 5 years in 2016, and the RV-associated mortality rate was highest in developing countries (Crawford et al., 2017; Troeger et al., 2018). RV surveillance from 2008 to 2018 found that 40.78% of all diarrheal disease in children in Southeast Asia was caused by RV infection (Lestari et al., 2020).

Rotaviruses are members of the family *Reoviridae*, the genus *Rotavirus*. They are classified into 10 species designated A through J, based on the antigenic properties of VP6 (Crawford et al., 2017). Human rotavirus A (RVA) is the most common etiological cause of severe gastroenteritis in young humans. The RV virion has an icosahedral symmetry with three concentric protein layers (i.e., an outer capsid, an inner capsid, and an internal core) that contains 11 double-stranded RNA (dsRNA) segments (McClain et al., 2010). Each segment is translated into six structural viral proteins (VPs) and six non-structural viral proteins (NSPs). The RV outer capsid consists of two neutralization antigens, VP7 and VP4, which are categorized into glycoprotein (G) and protease (P) types, respectively (Fujii et al., 2014).

The Rotavirus Classification Working Group (RCWG) has designated the genotype constellations for RVA strains for each of the 11 RV genome segments encoding *VP7–VP4–VP6–VP1–VP2–VP3–NSP1–NSP2–NSP3–NSP4–NSP5/6*, which corresponds to genotypes *Gx-P[x]-Ix-Rx-Cx-Mx-Ax-Nx-Tx-Ex-Hx*, respectively. Two major genotype constellations of the non-G, non-P genes; I1–R1–C1–M1–A1–N1–T1–E1–H1 (Wa-like) and I2–R2–C2–M2–A2–N2–T2–E2–H2, (DS-1-like), have been shown to circulate worldwide among humans (Matthijnssens et al., 2008). A third (minor) human genotype constellation, referred to as AU-1-like (I3–R3–C3–M3–A3–N3–T3–E3–H3), is believed to originate from cats or dogs (Nakagomi & Nakagomi, 1989).

Recently 36 G, 51 P, 26 I, 22 R, 20 C, 20 M, 31 A, 22 N, 22 T, 27 E and 22 H genotypes of RVA has been identified in humans and animals worldwide (RCGW, 2019). Some specific G and P genotypes are dominant in individual host species, six G genotypes (G1–G4, G9 and G12) and 3 P genotypes (P[4], P[6] and P[8]) are frequently found in humans. The G1P[8], G2P[4], G3P[8], G4P[8], G9P[8] and G12P[8] RVA strains were identified as the most prevalent G-P genotype combination worldwide (Crawford et al., 2017).

Presently, several commercial RV vaccines have been licensed for use. Two live attenuated oral vaccines, Rotarix^®^ (a monovalent G1P[8] attenuated human vaccine) and RotaTeq^®^ (a pentavalent human, bovine reassorted vaccine containing G1, G2, G3, G4 and P[8]) are administered routinely as part of the national immunization program (NIP) for the prevention of RV-associated severe gastroenteritis in developed countries (Vesikari, 2012). They have proven highly efficient in reducing the hospitalization of RV-associated severe gastroenteritis (Tate & Parashar, 2014). In Thailand, RVA is a significant cause of severe diarrhea in children (Theamboonlers et al., 2005). The surveillance in Thailand has indicated that the peak of RV infection corresponds to the winter months (December, January and February) (Maneekarn & Ushijima, 2000). In the northern part of Thailand, where the weather is relatively cold than the rest of the country, RV was detected in every month of the year (Sakpaisal et al., 2019). From 2009 to 2014, studies revealed that G1P[8], G2P[4] and G3P[8] were the most frequently detected genotypes in Thailand and the more uncommon human RV strains such as G3P[9], G4P[6], G5P[6], G8P[8], G9P[8], G12P[6] and G12P[8] were also detected in some regions of Thailand (Khananurak et al., 2010; Maiklang et al., 2012; Chieochansin et al., 2016). Starting in January 2020, universal RV vaccination for infants has been implemented in Thailand (Lestari et al., 2020).

In this study, we investigated the prevalence, genotype diversity and molecular characterization of human RVA circulating in children under 15 years of age with acute gastroenteritis (AGE) in Thailand between January 2016 and December 2019. This study provides useful data relative to the circulating RV genotypes in the pre-vaccine era in Thailand.

## Materials and Methods

### Study population and stool samples

Between January 2016 and December 2019, a total of 2,001 stool samples were collected from neonates to children under 15 years of age. The samples were obtained from the Chulalongkorn Memorial Hospital, Bangpakok 1 International Hospitals, Bangpakok 9 International Hospitals, Chumphae Hospital and Bangkok Hospital Phitsanulok. The enrolled patients’ presented with AGE characterized by three or more loose, watery stools within 24 h. The use of stored samples was approved by the director of King Chulalongkorn Memorial Hospital. The study was approved by the Ethics Committee of the Faculty of Medicine, Chulalongkorn University (IRB No. 220/63). The Institutional Review Board waived the need for consent from the participants because the clinical specimens were anonymous. The study was followed the declaration of Helsinki and Good Clinical Practice Guidelines (ICH-GCP).

### Molecular diagnosis of rotavirus

#### Viral genome extraction

Viral genome was prepared from a 10% (w/v) stool suspension with phosphate buffer saline (PBS), centrifuged at 4,000×*g* for 10 min, and supernatants were collected. Viral RNA was automatically extracted from a 200 µL supernatant sample using a magLEAD 12gC instrument (Precision System Science, Chiba, Japan) with a magLEAD Consumable Kit (Precision System Science, Chiba, Japan) according to the manufacturer’s instructions.

Samples were initially tested for the RVA VP6 gene using the QuantiTect SYBR Green 1-step real-time RT-qPCR Kit (Qiagen, Hilden, Germany). The primers VP6-F (5′ GACGGVGCRACTACATGGT 3′) and VP6-R (5′ GTCCAATTCATNCCTGGTGG 3′) a 379-bp region corresponding to nucleotides 747–1,126 of the VP6 gene. Cycling parameters were reverse transcription at 50 °C for 30 min, initial denaturation at 95 °C for 15 min, 45 cycles of denaturation at 94 °C for 15 s, annealing at 60 °C for 30 s, and extension at 72 °C for 30 s. Melting curve analysis was explored from 60 °C to 95 °C with 1 °C increments to determine the specificity of the reactions (Kang et al., 2004; Chansaenroj et al., 2020).

### Sequence determination of G, P and I genotype of rotavirus

The RV-positive sampleswere subjected to amplification of the VP6, VP7 and VP4 genes using the SensiFAST one-step RT-PCR kit (Bioline, London, UK). VP6-F1/VP6-R1357 from of Theamboonlers et al. (2005) were used to amplify the VP6 gene, and the primer pairs BEG9/END9 and con2/con3 were used to amplify VP7 and VP4 genes, respectively. Table S1 shows the list of primers that were used to detect the RV genotype. The total reaction mixture was 15 µl, consisted of a 2x reaction mix buffer, 10 µM of each forward and reverse primer and 2 µL RNA. The RT-PCR conditions for VP6 gene comprised the reverse transcription step at 45 °C for 45 min, followed by initial denaturation at 95 °C for 5 min, 40 cycles of denaturation at 94 °C for 30 s, annealing at 55 °C for 30 s, extension at 72 °C for 2 min, and the reaction concluded with a final extension at 72 °C for 10 min. The RT-PCR conditions of VP7 and VP4 genes were: reverse transcription at 45 °C for 30 min, followed by initial denaturation 94 °C for 5 min, 40 cycles of denaturation at 94 °C for 45 s, annealing at 48 °C (for BEG9 and END9) or 55 °C (for con2 and con3) for 30 s, extension at 68 °C for 1 min, and included a final extension step at 68 °C for 5 min (Chansaenroj et al., 2020). The expected amplicons were agarose gel-purified and sequenced by FirstBASE Laboratories (SDN BHD, Selangor, Malaysia).

The sequencing data of the VP6, VP7 and VP4 genes were analyzed using Chromas 2.23 (Technelysium, QLD, Australia). The nucleotide sequence identity was annotated by BLAST, and the rotavirus genotype was identified by using the RotaC^2.0^ automated genotyping tool for group A rotaviruses (RotaC version 2).

### Phylogenetic analysis

The nucleotide sequences were prepared and multiply-aligned using Clustal Omega (www.ebi.ac.uk/Tools). Phylogenetic trees and genomic distances were established using MEGA 6.0 software. The phylograms of VP6, VP7 and VP4 genes were constructed using the Tamura 3-parameter model, and the maximum likelihood method with 1,000 replicates bootstrapping and bootstrap values >70% considered significant.

Nucleotide sequences were deposited in the GenBank database under the accession numbers MW058089–MW058379 for VP7, MW058380–MW058573 and MW245377 for VP4 and MW058574–MW058799 for VP6.

## Results

**Figure 1:**
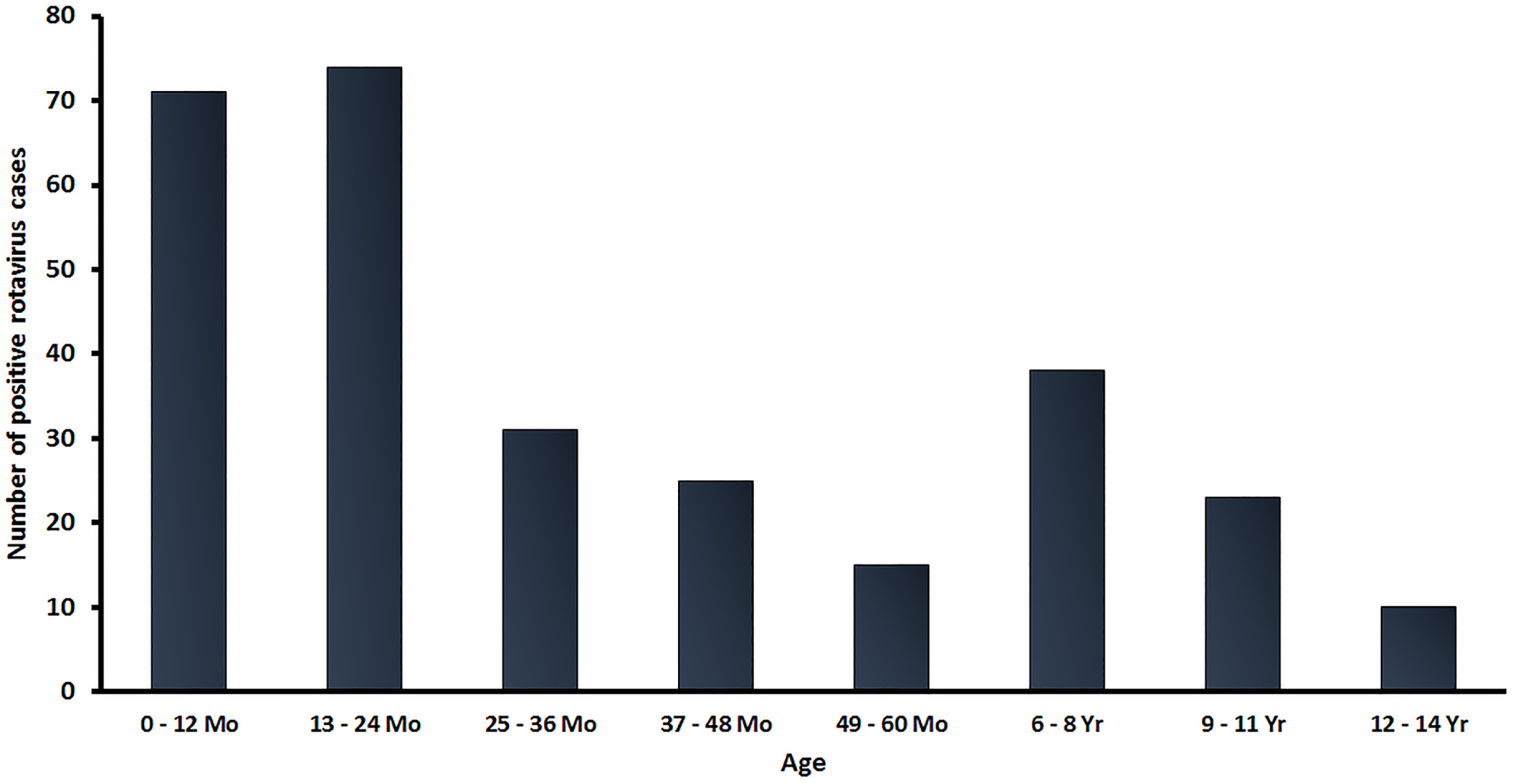
Age distribution of RVA infection among children in Thailand from January 2016 to December 2019. Bar graphs denote the total number of RV-positive samples.

In this study, a total of 2,001 stool samples were collected from children under 15 years of age diagnosed with AGE from 2016 to 2019. There were 438, 411, 857 and 295 stool samples in 2016, 2017, 2018 and 2019, respectively. Overall, 301 samples (15.0%) tested positive for RVA by real-time RT-PCR. The age distribution of rotavirus infected patients in this study ranged from neonate to children under 15 years of age, with the highest rate of RV-positive patients (48.2%; 145/301) occurring in children between 0 and 24 months of age (Fig. 1).

**Figure 2:**
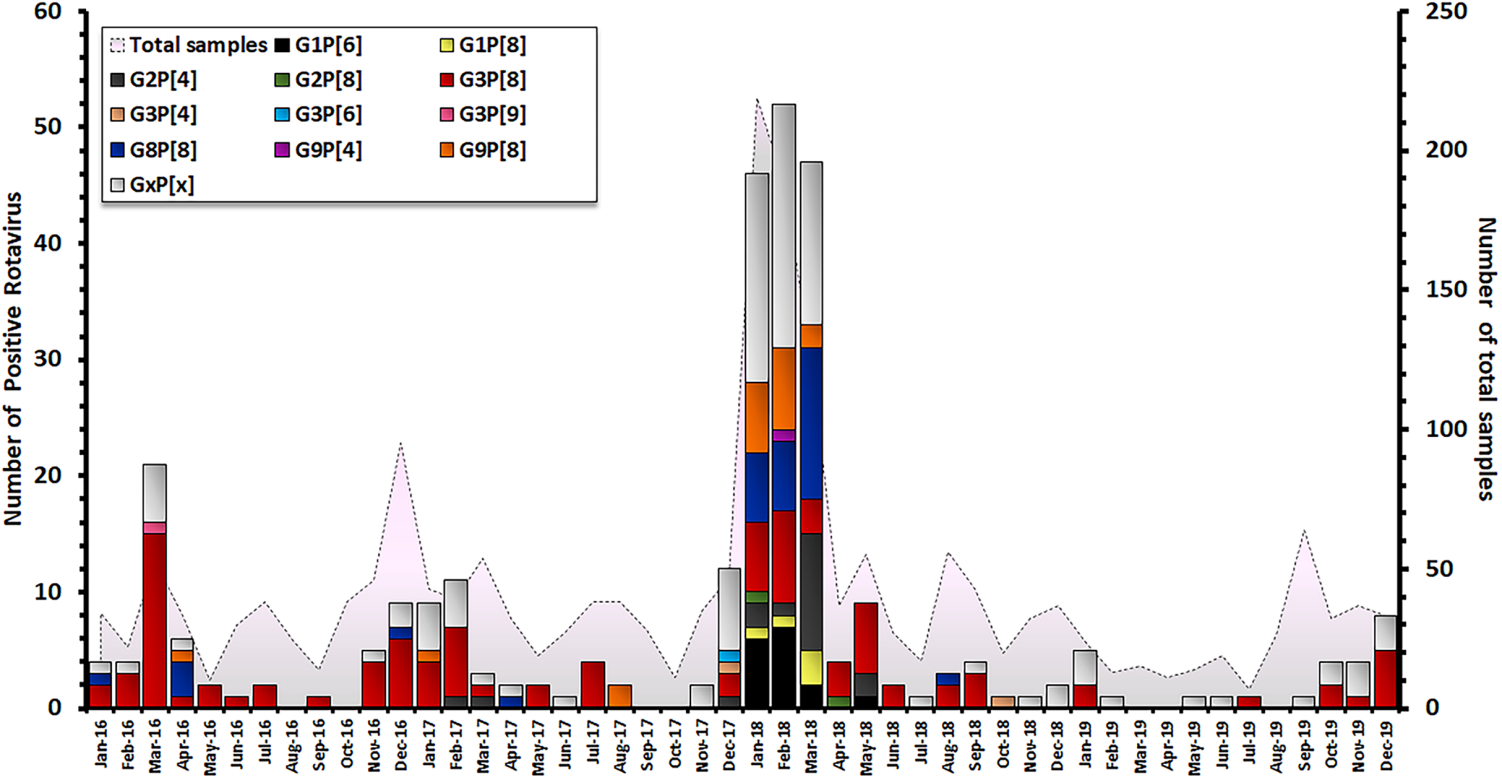
Distribution of RVA genotypes between January 2016 and December 2019. The monthly number of samples from children diagnosed with AGE is shown in grey. Bar graphs show the number of RV-positive cases.

The characterization of 301 RV-positive samples from children diagnosed with AGE in Thailand between 2016 and 2019 demonstrated that the G3P[8] genotype was the most dominant genotype (33.2 %, 100/301), followed by G8P[8] (10.6%, 32/301), G9P[8] (6.3%, 19/301), G2P[4] (6.0%, 18/301), and G1P[6] (5.3%, 16/301). Uncommon G and P combinations such as G2P[8] (0.7%, 2/301), G3P[4] (0.7%, 2/302), G3P[9] (0.3%, 1/301) and G9P[4] (0.3%, 1/301) were also detected at low frequencies (Table 1). The incidence of RV infection in Thailand decreased during the rainy season (June–October). Contrarily, an outbreak of RV infection was observed in during the winter season (December–March) and G3P[8] became the most predominant genotype every year. December 2017 to March 2018 marked the period with the highest prevalence of RV infection over a 4-year observation period, accounting for 157/301 of cases (Fig. 2).

**Table 1:** Distribution of RVA genotypes among children diagnosed with AGE in Thailand during 2016–2019.

Year	No. of specimens tested	No. of RVA positive (%)	No. of G/P combination strain (%)											
			G1P[6]	G1P[8]	G2P[4]	G2P[8]	G3P[8]	G3P[4]	G3P[6]	G3P[9]	G8P[8]	G9P[4]	G9P[8]	Untypable*
2016	438	55 (12.6)	0	0	0	0	37 (67.3)	0	0	1 (1.8)	5 (9.1)	0	1 (1.8)	11 (20.0)
2017	411	48 (11.7)	0	0	3 (6.3)	0	19 (39.6)	1 (2.1)	1 (2.1)	0	1 (2.1)	0	3 (6.3)	20 (41.7)
2018	857	172 (20.1)	16 (9.3)	5 (2.9)	15 (8.7)	2 (1.2)	33 (19.2)	1 (0.6)	0	0	26 (15.1)	1 (0.6)	15 (8.7)	58 (33.7)
2019	295	26 (8.8)	0	0	0	0	11 (42.3)	0	0	0	0	0	0	15 (57.7)
Total	2,001	301 (15.1)	16 (5.3)	5 (1.7)	18 (6.0)	2 (0.7)	101 (33.6)	2 (0.7)	1 (0.3)	0	32 (10.6)	1 (0.3)	19 (6.3)	104 (34.6)

**Note:**

* No amplicon/no PCR product/unsuccessful sequencing.

The sequencing of VP6 was performed to differentiate the I type and Wa, DS-1 and Au genogroups. The results showed that the unusual DS-1-like strain (G3/8/9-P[4/6/8]-I2) was the most prevalent (42.2%, 127/301), followed by Wa-like strain (G1/3/9-P[4/8]-I1) (13.0%, 39/301), DS-1-like strain (G2-P[4]-I2) (6.6%, 20/301), the Au-like strain (G3-P[9]-I3) (0.3%, 1/301), and unclassified types (38.2%, 115/301) (Table 2).

**Table 2:** Distribution of G/P/I combination strain of RVA genotypes among children diagnosed with AGE in Thailand during 2016–2019.

Year	No. of specimens tested	No. of RVA positive (%)	No. of G/P/I combination strain (%)				
			Unusual DS-1-like (G1/3/8/9-P[4/6/8]-I2)	DS-1-like (G2-P[4]-I2)	Wa-like (G1/3/9-P[4/8]-I1)	Au - like (G3-P[9]-I3)	Unclassified
2016	438	55 (12.6)	34 (61.8)	0	1 (1.8)	1 (1.8)	19 (34.5)
2017	411	48 (11.7)	20 (41.7)	3 (6.3)	3 (6.3)	0	22 (45.8)
2018	857	172 (20.1)	64 (37.2)	17 (9.9)	32 (18.6)	0	59 (34.3)
2019	295	26 (8.8)	9 (34.6)	0	2 (7.7)	0	15 (57.7)
Total	2,001	301 (15.1)	127 (42.2)	20 (6.6)	38 (12.6)	1 (0.3)	115 (38.2)

**Figure 3:**
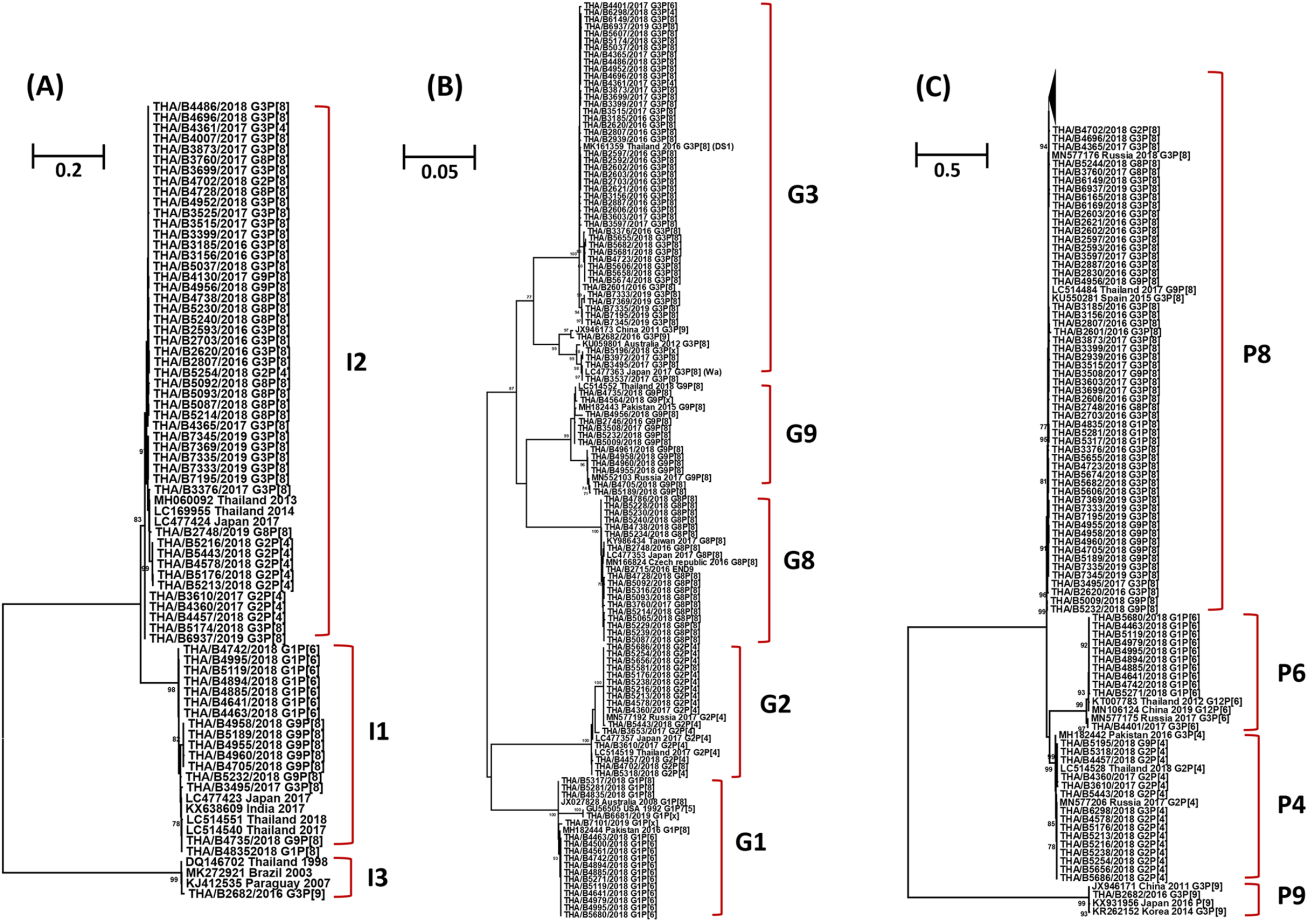
Phylogenetic tree of the VP6, VP7 and VP4 genes. The tree was constructed using the Tamura 3-parameter model, and the maximum likelihood method with 1,000 replicates bootstrapping and bootstrap values >70% considered significant. (A) Partial VP6 gene analyzed in this tree contained 361 nucleotides, (B) partial VP7 gene analyzed in this tree contained 561 nucleotides and (C) partial VP four gene analyzed in this tree contained 438 nucleotides.

The phylogenetic tree analysis of the VP6 gene (I type) segregated into three groups, the Wa-like strains (I1) shared 96.3-100% nucleotide identity, the DS-1-like strains (I2) shared 95.7–100% identity, and one Au-like strain (I3) showed closer genetic relatedness to the RVA strain (KJ412535) previously detected in Paraguay in 2007, with nucleotide and amino acid similarities of 98.7% (Fig. 3A).

Based on the sequence of the VP7 gene, the majority of G1 strains were similar to strains JX027828 and MH182444 from Australia and Pakistan, respectively, with 92.8–100% nucleotide identity. In addition, one strain of the G1 genotype was closely related to the RotaTeq^®^ vaccine strain (GU565057) with nucleotide similarity of 100%. Analysis with a phylogenetic tree for the G2 genotype indicated the samples shared nucleotide and amino acid identity exceeding 95%. Interestingly, the phylogenetic tree analysis of the G3 genotype showed a different segregation of Wa-like G3 and unusual DS-1-like G3, with a nucleotide identity ranging from 80.7% to 82.3%. The analysis of G8 strains indicated they shared 98.7–100% identity and exceeded 98.9% with the reference strains LC477353, KY986434 and MN166824 from Japan, Taiwan, and the Czech Republic, respectively. For the G9 genotype, the strains shared a 92.3–100% nucleotide identity and 92.6–99.8% nucleotide identity to the reference strains MN552103 and MH182443 from Russia and Pakistan, respectively (Fig. 3B).

Three P genotypes were identified in this study, the predominance of the P[8] genotype showed there was a 96.3–100% nucleotide identity across the genotypes, and exceeded 95.8% with the reference strains MN577176 from Russia, KU550281 from Spain and LC514484 from Thailand. The P[6] strains shared 92.6–100% nucleotide identity and were similar to the reference strains MN106124 from China, exceeding 93.6% nucleotide identity. The P[4] strains shared 96.3–100% nucleotide identity among their strains and exceeded 96.0% with the reference strains MN577206, MH182442 and LC514528 from Russia, Pakistan and Thailand, respectively. In addition, one strain of the P[9] genotype was closely related to reference strains JX946171, KX931956 and KR262152 from China, Japan and Korea, respectively, with 98.0–99.1% nucleotide identity (Fig. 3C).

## Discussion

Rotavirus A infection is a major cause of severe diarrhea in Thai children. Between 2007 and 2014, the prevalence of RVA infection in Thailand was found to range from 28.4 to 44.5% (average 34.3%) (Khananurak et al., 2010; Maiklang et al., 2012; Chieochansin et al., 2016). In this study, we investigated the prevalence and distribution of RVA genotypes circulating in children under 15 years of age diagnosed with AGE in Thailand from 2016 to 2019. Among the 2,001 stool samples obtained from infants and young patients with AGE over a 4-year study period (January 2016 to December 2019), 301 samples (15.0%) tested positive for RVA. Interestingly, there was a large-scale outbreak of RVA infection in both children and adult between December 2017 and March 2018 (Chansaenroj et al., 2020). The highest frequency of RVA infection in this study occurred in children aged 0–24 months (48.2%), which corresponded to data from a previous study in Mexico, in which almost all of the children by 2 years of age had been infected at least once, and more than two-thirds had experienced at RVA least twice, and more than 10% had experienced five infections (Velázquez et al., 1996). However, in older children there was a clear reduction in RVA infection. A possible explanation for this could be the acquisition of protective immunity in older children during prior exposure to RV, then they became resistant to a subsequent RVA infection (Echeverria et al., 1983; Linhares et al., 1989). In addition, the rate of RVA infection increased over the winter season in Thailand (December–March), which corresponded to the seasonal pattern of RVA infection in Thailand, as described in a previous surveillance study (Maiklang et al., 2012).

In the surveillance of G/P genotype distributions in Thailand between 2000 and 2016, various G/P genotype combinations were detected. The common G1P[8], G2P[4], G3P[8] and G9P[8], and uncommon G2P[8], G3P[3], G3P[9], G3P[10], G3P[19], G12P[6] and G12P[8]. Moreover, a changing pattern of RV genotypes was observed. The G9P[8] had been reported to be the most predominant genotype from 2000 to 2004, G1P[8] from 2005 to 2009, G3P[8] from 2009 to 2011, G1P[8] from 2012 to 2014 and G9P[8] from 2015 to 2016, and more uncommon genotypes such as G12P[8], G12P[6] and G3P[9] were also detected (Pongsuwannna et al., 2010; Maneekarn & Khamrin, 2014; Theamboonlers et al., 2014; Guntapong et al., 2017; Chan-It & Chanta, 2018). In this study, G3P[8] was the most predominant G/P genotype combination (33.6%, 101/301), followed by G8P[8] (10.6%, 32/301), G9P[8] (6.3%, 19/301), G2P[4] (6.0%, 18/301) and G1P[6] (5.3%, 16/301). Moreover, G1P[8], G2P[8], G3P[4], G3P[6], G3P[9] and G9P[8] were found in low frequency. In the genetic backbone characterization, the DS-1-like strain was detected in 147 of 301 samples (48.8%), most of which were unusual DS-1-like G3P[8]I2 (57.8%, 85/147), followed by unusual DS-1-like G8P[8]I2 (21.1%, 31/147) and G2P[4]I2 (12.2%, 18/147).

Since 2012, the more uncommon human intergenogroup reassortant strains DS-1-like G1P[8], were firstly detected in children with severe diarrhea in Japan (Fujii et al., 2014; Kuzuya et al., 2014; Maneekarn & Khamrin, 2014; Yamamoto et al., 2014), and subsequently, these strains were also identified in Thailand, the Philippines, Vietnam and Brazil between 2012 and 2013 (Yamamoto et al., 2017; Komoto et al., 2015; Nakagomi et al., 2017; Luchs et al., 2019). In 2013, the DS-1-like G3P[8] strain emerged in Thailand and Australia (Cowley et al., 2016; Komoto et al., 2016), and these DS-1-like G3P[8] strains were subsequently detected in the several parts of the world, comprising Hungary, Germany, Indonesia, Thailand, Japan, Spain, USA, Brazil and Italy (Arana et al., 2016; Dóró et al., 2016; Guerra et al., 2016; Komoto et al., 2016; Kikuchi et al., 2018; Komoto et al., 2018; Pietsch & Liebert, 2018; Utsumi et al., 2018; Esposito et al., 2019). Furthermore, in this study, the unusual DS-1-like G3P[8] strains were identified as the most predominant strains in infants and young children in Thailand. In the phylogenetic trees analysis, the VP7 gene of these unusual DS-1-like G3P[8] strains were found to be closely related with the reference strain MK161359 from Thailand in 2016, with 98.0–99.8% nucleotide identity. In addition, the VP4 and VP6 genes of these strains also shared a high percentage of nucleotide similarity with the unusual DS-1-like backbone from Spain, Russia, Japan, and Thailand. Therefore, these unusual DS-1-like G3P[8] strains isolated in different locations in Thailand might have originated from a recent common origin. These unusual DS-1-like G3P[8] strains likely were generated in Thailand and then circulated in this country, or even they were transmitted from another country to Thailand. However, our study was constrained by the limited sequencing information from clinical samples because they sometimes did not yield sufficiently long sequences to provide conclusive support for genotyping. Unfortunately, it is difficult to extract and amplify enough viral genetic materials for characterization due to viral loads and time of sampling. Therefore, it is possible that more complete sequence results may alter our conclusion in this study. More genome data for global RVA strains are required to gain a better understanding of the evolution of DS-1-like G3P[8] strains.

In industrialized countries, rotavirus genotype G8 infection is common in cattle but has been identified in humans sporadically (Moutelíková et al., 2019). However, G8 strains are highly prevalent among humans in some countries in Africa (Nakagomi et al., 2013) and were also described in Brazil and Chile (Santos et al., 1998; Gómez et al., 2010; Lucero et al., 2019). During the past few years, reports of G8 rotavirus-strain detection have been increasing. Between 2013 and 2014, the novel DS-1-like intergenogroup reassortant G8P[8] were firstly detected in stool samples from hospitalized children with severe diarrhea in Thailand, and these DS-1-like G8P[8] strains were subsequently detected in Japan in 2014, Vietnam in 2015, and Czech Republic in 2016/2019 (Tacharoenmuang et al., 2016; Kondo et al., 2017; Hoa-Tran et al., 2016; Moutelíková et al., 2019). Moreover, in this study, the DS-1-like G8P[8] became the second most prevalent genotype. Therefore, the high prevalence of DS-1-like G8P[8] strains that were described in this study and other countries indicates that these strains are well-adapted to human–human transmission. The continuous surveillance of the genotypes of RVA isolates is recommended in order to monitor circulating wild-type strains, as well as rotavirus genotype constellations, to understand rotavirus diversity and their evolutionary patterns.

In the pre-vaccine era G1P[8], G2P[4], G3P[8], G4P[8] and G9P[8] represented approximately 74% of strains causing RV infections in 1996–2008 (Bibera et al., 2020). The introduction of RotaTeq^®^ was followed by an increase in the prevalence of the G3P[8] genotype in some places, including the United States and in some Australian states (Hull et al., 2011; Kirkwood et al., 2011; Cowley et al., 2016). An increase in the relative prevalence of the fully heterotypic G2P[4] genotype occurred after the introduction of Rotarix^®^ (Dóró et al., 2014), particularly in Brazil, Belgium and in some Australian states. It raised concern about a potential selection pressure induced by vaccine use. Typically, G1P[8] strains, including the Rotarix^®^ strain (RIX4414), possess a Wa-like genetic backbone, whereas G2P[4] strains generally have a DS-1-like genotype constellation (Matthijnssens et al., 2011). In a later trial conducted in Europe (Vesikari et al., 2007) and a meta-analysis integrating the results from all previous trials (De Vos et al., 2009), *the* Rotarix^®^ vaccine provided significant protection against severe RV diarrhea caused by G2P[4] strains. Annual fluctuations in G2P[4] prevalence seemed to occur naturally, with no substantial differences between countries adopting Rotarix^®^, RotaTeq^®^, or mixed vaccination programs (Matthijnssens et al., 2011). Before January 2020, RV vaccine was not included in the national immunization program in Thailand but only introduced in two provinces, Sukhothai and Petchabun. In October 2011, Sukhothai province began a routine RV immunization program. The evaluation for first introduction was done in 2017. It was concluded that RV vaccine was highly effective to prevent diarrhea and provide herd immunity among children who had not been vaccinated (Tharmaphornpilas et al., 2017). These findings support the continued use of the RV vaccine as an intervention to reduce severe diarrhea caused by RV strains possessing either Wa-like or DS-1–like genetic backbones.

## Conclusions

The surveillance of RVA infection in infants and young children diagnosed with AGE in Thailand between 2016 and 2019 demonstrated that RVA occurred most frequently primarily among children aged 0–24 months. The increasing detection of RVA infection during winter months significantly correlated to the seasonal pattern of rotaviruses in Thailand, as described in a previous surveillance study (Maiklang et al., 2012). The unusual DS-1-like G3P[8] was identified as the most predominant genotype. A review of global changes in the overall RV strain prevalence did not show any consistent patterns of selection pressure resulting from the use of either Rotarix^®^ or RotaTeq^®^ vaccines (Dóró et al., 2016). Thus, it is important to continue surveillance of rotavirus epidemiology and rotavirus characterization to obtain useful information for the prevention and control of RVA, and to gain a better understanding of the effects of strain variation on vaccine efficacy.

## Supplemental Information

10.7717/peerj.10954/supp-1 Supplemental Information 1 Raw data.

10.7717/peerj.10954/supp-2 Supplemental Information 2 Primers for RT-PCR detection of Rotavirus Genotype.

## Additional Information and Declarations

### DNA Deposition

The following information was supplied regarding the deposition of DNA sequences: Raw data are available at NCBI: MW058089–MW058379 (VP7); MW058380–MW058573 and MW245377 (VP4); and MW058574–MW058799 (VP6).

### Data Availability

The following information was supplied regarding data availability: Raw data are available in the Supplemental Files.
