# Supplementary material for: High prevalence of circulating DS-1-like human rotavirus A and genotype diversity in children with acute gastroenteritis in Thailand from 2016 to 2019
Source: PeerJ. 2021 Feb 26;9:e10954. doi: 10.7717/peerj.10954 (PMC7919534; doi:10.7717/peerj.10954)
Supplement: Supplemental Information 2 [file peerj-09-10954-s002.docx]

S1. Primers for RT-PCR detection of Rotavirus Genotype

| Segment | Primer | Sequence | Position |
| --- | --- | --- | --- |
| *VP7* | *BEG9* | 5’- GGCTTTAAAAGAGAGAATTTCCGTCTGG -3’ | *1-28* |
|  | *END9* | 5’- GGTCACATCATACAATTCTAATCTAAG - 3’ | *1036-1062* |
| *VP4* | *CON3* | 5'- TGG CTT CGC CAT TTT ATA GAC A -3' | *11-32* |
|  | *CON2* | 5'- ATT TCG GAC CAT TTA CC-3' | *868-887* |
| *VP6* | *ROTA_VP6_F1* | 5’– GGCTTTAAAACGAAGTCTTC -3’ | *1-20* |
|  | *ROTA_VP6_R1357* | 5’– GGTCACATCCTCTCACTA -3’ | *1338-1357* |
